# The Effects of Ageing and Visual Field Loss on Pointing to Visual Targets

**DOI:** 10.1371/journal.pone.0097190

**Published:** 2014-05-16

**Authors:** Nikki J. Rubinstein, Andrew J. Anderson, Anna Ma-Wyatt, Mark J. Walland, Allison M. McKendrick

**Affiliations:** 1 Department of Optometry & Vision Sciences, The University of Melbourne, Parkville, Australia; 2 School of Psychology, The University of Adelaide, Adelaide, Australia; 3 Victoria Parade Eye Consultants, Fitzroy, Australia; Harvard Medical School, United States of America

## Abstract

**Purpose:**

To investigate the effect of ageing on visuomotor function and subsequently evaluate the effect of visual field loss on such function in older adults.

**Methods:**

Two experiments were performed: 1) to determine the effect of ageing on visual localisation and subsequent pointing precision, and 2) to determine the effect of visual field loss on these outcome measures. For Experiment 1, we measured visual localisation and pointing precision radially at visual eccentricities of 5, 10 and 15° in 25 older (60–72 years) and 25 younger (20–31 years) adults. In the pointing task, participants were asked to point to a target on a touchscreen at a natural pace that prioritised accuracy of the touch. In Experiment 2, a subset of these tasks were performed at 15° eccentricity under both monocular and binocular conditions, by 8 glaucoma (55–76 years) and 10 approximately age-matched controls (61–72 years).

**Results:**

Visual localisation and pointing precision was unaffected by ageing (p>0.05) and visual field loss (p>0.05), although movement time was increased in glaucoma (p = 0.01).

**Conclusion:**

Visual localisation and pointing precision to high contrast stimuli within the central 15° of vision are unaffected by ageing. Even in the presence of significant visual field loss, older adults with glaucoma are able perform such tasks with reasonable precision provided the target can be perceived and movement time is not restricted.

## Introduction

Vision is a key sensory input used in planning visually guided hand movements. For example, it is difficult to reach for a fallen object from the floor without first locating it visually. The initial visual position information used to localise the target (or goal) of a reach plays an important role in determining the endpoint precision (a measure of variability, as distinct from endpoint bias) of the reaching movement, as evidenced by studies of rapid pointing to visual targets (movement times of 400–500 ms) [1], [2]. When there is large uncertainty in the initial visual position estimate of the target, visual error limits the endpoint precision of pointing movements [1]. When visual localisation and pointing judgements are measured for a single stimulus, the location error in these two measures also shows 60% agreement across subjects using a trial-by-trial analysis [2]. Because action can be limited by visual information, if visual localisation is impaired through disease or ageing there is the potential for related changes in pointing behavior and goal directed hand movements.

Glaucoma is a progressive optic neuropathy that primarily affects older adults [3] and is the second leading cause of irreversible blindness in developed nations [4]. Recent work shows that visually guided hand movements are altered in individuals with glaucoma [5], presumably due to their reduced contrast sensitivity in the peripheral visual field (visual field loss). Although visual field loss due to glaucoma can progress rapidly, it is more common for damage to slowly progress over years to decades [6]. Because of this, behavioural performance in glaucoma might not simply reflect flow-on effects of difficulty seeing peripheral targets, but also the effects of compensatory processes available within the ageing visuomotor system.

Kotecha et al. [5] found that, although glaucoma did not affect grip size at object contact in grasping movements, patients with glaucoma made more tentative goal-directed reaching movements than age-matched normally-sighted controls. These tentative movements were suggestive of an impairment of initial movement planning and control and were not related to the overall amount of visual field loss. It is not known whether patients were less precise in guiding hand movements in addition to being more tentative, however. Given that glaucoma affects fine spatial localisation judgements in foveal vision [7], there is also the possibility of a more general loss of visual localisation precision in glaucoma that might impact upon the ability to guide pointing behaviour. Whether any such loss of localisation precision would be predictable from conventional behavioural tests for glaucoma that measure contrast sensitivity to small spots of light across the visual field (static automated perimetry), is not clear.

Several studies have explored goal directed movements in normally sighted older adults [8], [9], but not with the specific intent of comparing these movements to possible changes in visual performance. In older adults, reaction times increase [8] and the precision of pointing movements reduces when constrained to a single plane of movement [9]. Older adults also spend longer in phases of acceleration and deceleration, reaching a slower peak velocity than younger adults during a reach [10]–[12]. Rossit and Harvey [13] have shown that, although the ability to perform corrective movements is intact in older adults, there is a general slowing of the planning, initiation and execution aspects of pointing movements. Older participants require 50% longer than younger participants for sensory feedback information to impact on the trajectory of a reach [14]–[16], suggesting that sensorimotor integration is altered with normal ageing. Whether this is due to slower visual processing or slower visual feedback integration is unknown.

In the current study, we make a direct comparison of visual localisation and pointing precision in younger and older adults. By quantifying performance on a visual localisation task and measuring pointing performance to similar stimuli, we attempt to identify whether motor precision is directly affected by any effects of ageing to visual localisation precision or whether the motor system is able to compensate and maintain performance.

We also examine performance in a group of older adults with clearly degraded sensory performance, as a result of visual field loss from glaucoma. We aimed to determine whether having reduced visual field sensitivity, as assessed by static automated perimetry, is related to either visual spatial localisation ability and/or subsequent pointing precision. We tested participants monocularly (the standard clinical method of assessing vision), and also binocularly as this is the habitual situation for visuomotor tasks in daily life.

## Materials and Methods

The study was approved by the Human Research Ethics Committee (HREC) of The University of Melbourne and complied with the tenets of the Declaration of Helsinki. Participants provided written informed consent prior to participation according to the protocol approved by our HREC.

### Participants

The study comprised of two experiments: 1) a comparison of younger adults to older adults and 2) a comparison of older adults with glaucoma to a group of approximately age-matched controls.

The first experiment included 25 younger (aged 20 to 31 years, mean 25 years, 8 male) and 25 older (aged 60 to 72 years, mean 67 years, 9 male) participants. One older male participant was excluded because his movement times were more than 5 standard deviations (SDs) slower than the average recorded movement time for the remainder of the older adults. The second experiment tested ten normally-sighted older adults (aged 61 to 72 years, mean 68 years, 3 male) and eight older adults with glaucoma (aged 55 to 76 years, mean 67 years, 3 male). Normally sighted participants were recruited via advertisements within The University of Melbourne and community centres, while glaucoma patients were recruited through the Australian College of Optometry and via the clinic of one of the authors (MW). All control participants underwent an eye examination to ensure that they met the following inclusion criteria: visual acuity better than 6/12 (20/40), refractive error ≤±5.00 D spherical and ≤2.00 D astigmatism, normal ocular health for age, normal visual fields as measured by the Central Fast Threshold testing paradigm on the Medmont M700 standard automated perimeter (a contrast sensitivity test to 0.43° luminous spots presented at defined locations across the visual field: Medmont Studio v 2.3, Medmont International Pty Ltd, Australia), and functional binocular vision (Worth-4-dot test at 40 cm and ≤110 seconds on the Frisbee stereotest) [17], [18]. Individuals in the glaucoma group were required to have an ophthalmological diagnosis and were being treated for glaucoma. All participants scored ≥24 on the Mini-Mental State Examination, indicating that there were no gross deficits in cognitive function [19]. Exclusion criteria for all groups included medications or systemic conditions known to affect vision or cognitive function, and medically treated arthritis. Participants completed the experiments in 2–3 sessions of up to 2 hours duration each.

### Experimental set-up

We displayed stimuli on a 17 inch (1024×768 pixels, 60 Hz) LCD touchscreen monitor (1729L; EloTouchSystems, Fremont, CA) at a viewing distance of 40 cm. Experimental software was custom-written in Matlab R2008a (Mathworks, Natick, MA, USA), using the Psychophysics Toolbox (v2.54) [20], [21]. IntelliTouch software (EloTouchSystems, Fremont, CA) determined the x,y co-ordinates and time when the finger lifted from the screen, and so the pointing times reported here will be longer and not directly comparable to studies where the time to when the screen was first touched is reported[1], [2], [22]. Head position was maintained with a chin rest.

Both experiments included a visual localisation and a pointing task. In Experiment 1 all tasks were performed binocularly, while in Experiment 2 tasks were performed binocularly (in order to more closely mimic real world functionality) and then monocularly (the typical method of clinically assessing visual performance). We assigned the monocular eye at random in controls (6 dominant eyes) and based on the presence of visual field damage at test locations in those with glaucoma (5 dominant eyes). We specifically aimed to test in areas of visual field loss. Tasks were self-paced with rest breaks as required.

### Visual localisation tasks

In a natural environment, targets (e.g. a cup on a desk) are often located close to other objects. Visual localisation under these circumstances may be improved compared to situations in which there is no reference (a target presented in isolation for example, swatting a fly on a blank wall). We therefore measured visual localisation with and without visual references.

**Figure 1:**
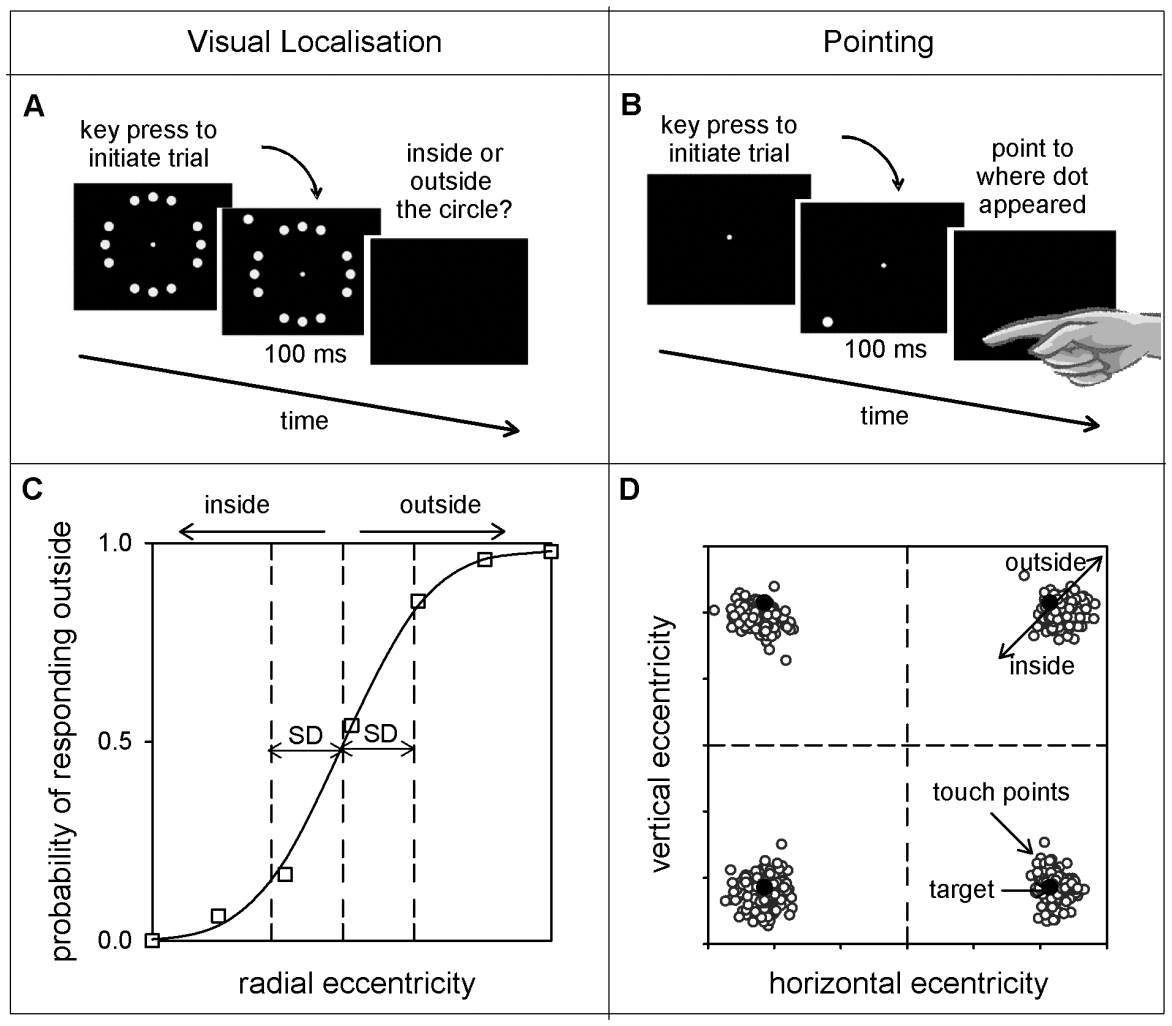
Schematic of the methods. Testing paradigm (referenced visual localisation, Panel A; pointing, Panel B) and example data for a single observer (and for the case of Panel D, a single quadrant). Stimuli were presented for 100 ms after the button press that initiated each trial. For the visual localisation task (Panel A), the target spot appeared at one of 28 (7 MOCS steps x 4 isoeccentric locations) possible target locations. For the pointing task (Panel B), the target appeared at one of 4 possible target locations. Pointing precision was calculated as the reciprocal of the standard deviation (SD) of the population of pointing errors in the radial direction (Panel D), and visual localisation precision calculated as the reciprocal of the SD of the psychometric function fitted to the frequency of responding that the spot appeared outside the reference circle (Panel C). We also performed an unreferenced visual localisation task (not shown) wherein the reference circle (12 circularly arranged target spots, with 4 gaps) was displayed only once at the beginning of a block of trials.

For the referenced visual localisation task, the task began with the presentation of a reference circle, whose radius corresponded to the tested eccentricity (5, 10 or 15°). The circle comprised 12 reference spots (white, 174 cd/m^2^, subtending 0.5° visual angle on a black background of 0.1 cd/m^2^), with gaps at the oblique axes of the circle corresponding to four possible target locations (Figure 1A). Participants fixated a central fixation point (grey, 40 cd/m^2^, subtending 0.25° visual angle) and pressed a key to initiate a trial. A target spot (white, 174 cd/m^2^, 0.5° visual angle) was presented for 100 ms at one of the four test locations. The target spot was of equivalent luminance to a 2.6 dB test stimulus using the dB scale used to present Medmont perimetry data. The radial position of the target was varied to measure a psychometric function using a method of constant stimuli (MOCS). Seven positions – one at the tested eccentricity, three farther and three closer to centre – were presented at each target location. Participants judged whether the target appeared farther out or closer in towards fixation than the tested eccentricity (reference circle), responding via a button press. Auditory feedback was given for incorrect responses. Step sizes were determined for each individual during task familiarisation, and were not statistically significantly different between groups in either experiment (Experiment 1: unreferenced – young mean 0.12, old mean 0.14, F(1,48)  = 2.17, p = 0.15, referenced – young mean 0.06, old mean 0.06, F(1,48)  = 0.87, p = 0.36; Experiment 2: old mean 0.054, glaucoma mean 0.075, F(1, 16)  = 3.84, p = 0.07). A schematic representation of the testing paradigm is shown in Figure 1A.

The unreferenced visual localisation task used the same testing paradigm as the referenced task (Figure 1A), except that the reference circle was only presented at the beginning of each block of trials and then disappeared. Observers were encouraged to begin as soon as the circle appeared, and so typically viewed the reference circle for only a few seconds during experimental runs, although the reference circle was constantly displayed for closer to a minute during task instruction at the beginning of the experiment. This initial presentation was to help observers form an internal representation of the tested eccentricity. Participants were required to make visual judgements relative to the remembered circle position. Auditory feedback was provided for incorrect responses to aid visual localisation and help participants maintain their criterion.

Visual localisation precision was defined as the reciprocal of the standard deviation of the cumulative Gaussian used to fit the psychometric function for the frequency of responding outside (Figure 1C). Psychometric functions were created using a maximum likelihood fit, and false positive and negative rates incorporated according to Abbott's formula [23]. Functions with less than two data points lying within the central 90% of the psychometric function range along the y-axis were excluded from further analysis (no functions were excluded from Experiment 1; 2 glaucoma participants and 2 older controls each had one quadrant rejected in Experiment 2), as well as quadrants corresponding to those discarded due to insufficient pointing data (1 glaucoma quadrant, described below).

### Pointing task

The pointing task was analogous to the unreferenced visual localisation task except participants were required to touch the target position (Figure 1B). A small amount of radial jitter was added to the targets, chosen randomly from a uniform distribution ranging ±10% of the tested eccentricity. We instructed participants to touch the position that had been occupied by the target spot on the touchscreen, using the same finger of their dominant hand that had been used to press the button that initiated the trial. The button was placed centrally on the table 5 cm in front of the chin rest, with movements from here to the screen ranging in amplitude from 30–45 cm. Hand dominance was determined by the Edinburgh Handedness Inventory [24]. Twenty-two of the younger participants and 20 of the older participants in Experiment 1 and seven of the older participants and all eight of the glaucoma participants in Experiment 2 were right handed.

Participants fixated the central spot at the beginning of each trial, but were free to make eye movements during the pointing movement in order to better mirror real-world functioning. As targets were only presented for 100 ms, any visually-driven saccades were executed after target offset [25]–[27]. Because we were interested in naturalistic behaviour, tasks were self-paced with no time restrictions applied for the movement. However, participants were asked to use a single smooth movement and aim for as close to the target position as possible. No feedback was given for movement accuracy or movement time, so as not to discourage any individuals with naturally poor pointing precision.

**Figure 2:**
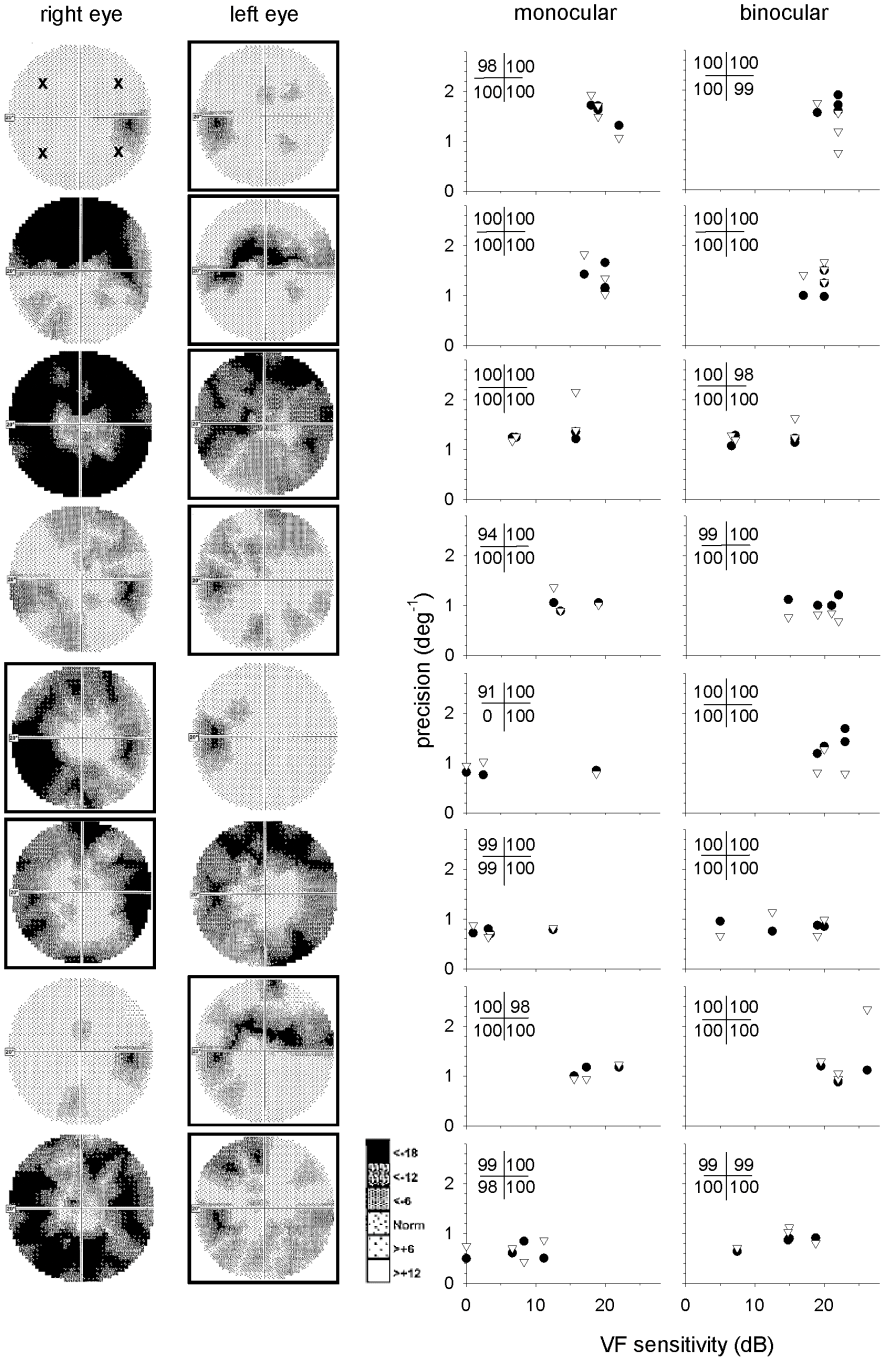
Visual fields and results for glaucoma participants in Experiment 2. Greyscale age matched normal visual field plots are shown on the left and precision plots on the right for each of the eight glaucoma patients. On the greyscale plots, the eye that was used for monocular testing has a border. The location of test stimuli are illustrated by the *x* symbols in the top-left greyscale panel. The precision plots show monocular (left) and binocular (right) visual localisation (•) and pointing (▿) precision as a function of visual field sensitivity for each test location. The numbers in the top left corner of each panel represent the percentage of pointing data that was used in the analysis. If less than 50% of trials were to the wrong quadrant, all data for that quadrant was excluded from analysis, as seen in row 5. The locations of the numbers correspond to the appropriate quadrants.

For each of the four locations around the circle we calculated the distance between the touched location and the target location in the radial direction for each trial, with pointing closer in being negative and pointing farther out positive (Figure 1D). Each of these error measures was pooled into a population for each quadrant, of each eccentricity, for each participant. Pointing precision was taken as the reciprocal of the SD of each population of errors. Pointing trials that were to the wrong quadrant were rejected from the analysis (less than 1% of trials in controls). A quadrant was excluded from further analysis if >50% of the touch points were outside the tested quadrant, as occurred for one glaucoma participant in one quadrant (see Figure 2, row 5, monocular condition).

### Experiment 1

The aim of Experiment 1 was to investigate how ageing affects the way in which visual localisation precision relates to pointing movement endpoints. In order to modulate difficulty, tasks were performed at 3 different eccentricities from fixation: 5°, 10° and 15° visual angle. Each participant sat 3 visual localisation blocks, of 112 trials each, for each eccentricity and visual localisation task (referenced and unreferenced). Two blocks of 80 trials were run for each eccentricity in the pointing task. The order of the runs was randomised across eccentricity and task and counterbalanced between groups. Quadrant data was pooled for each individual to give a single estimate of precision for each task.

### Experiment 2

The aim of Experiment 2 was to investigate how loss of visual field sensitivity affects the relationship between visual localisation precision and pointing movement endpoints. Only the referenced visual localisation and pointing tasks were included for this experiment. The referenced visual localisation task was chosen to increase the chance of detecting a difference, as Experiment 1 showed the referenced task to have less inter-observer variability in psychometric function slopes than the unreferenced condition. Testing was performed at 15° eccentricity only, first binocularly and then monocularly. An eccentricity of 15 degrees was chosen to test the region where glaucomatous visual field loss typically occurs. Quadrants were used to generate separate data sets in the analysis of Experiment 2. To do this, more trials were required, so the parameter set for this experiment was pared down to keep testing time reasonable. For each condition, 6 blocks of 112 visual localisation trials were interleaved with 4 blocks of 80 pointing trials.

### Visual field assessment

We measured visual fields with the central fast threshold paradigm on the Medmont M700 standard automated perimeter (Medmont Studio v 2.3, Medmont International Pty Ltd, Australia) [28]. Reliability indices (false positive, false negative and fixation loss) were ≤25% for all participants, and all control participants had global indices within the one-tailed 95% range of the Medmont normative database. In Experiment 2, the anti-logs of the absolute visual field sensitivities (dB) nearest 45°, 135°, 225° and 315° at an eccentricity of 15° visual angle from fixation were averaged to obtain estimates of visual field sensitivity at the target locations used (see top-left panel of Figure 2 for approximate target position) [29]. We assumed visual field sensitivity for binocular viewing conditions was equivalent to the best monocular sensitivity at that location, as per the method described by Nelson-Quigg, Cello and Johnson [30].

### Statistical analysis

Repeated measures analyses of variance (ANOVA) in SPSS [31] were used to test for differences in precision between the younger and older groups in Experiment 1. Two ANOVAs were performed separately for the visual localisation tasks and the pointing task. These analyses included both the between subjects factor of group (younger and older) and the within subjects factor of eccentricity (5, 10 and 15). Additionally, the analysis of visual localisation included a within subjects factor of task (referenced and unreferenced visual localisation).

Four separate 1-way ANOVAs were used to test for a difference in precision between glaucoma patients and age-matched controls in Experiment 2. The precision value for each individual was taken from the quadrant with the worst visual field sensitivity (lowest dB), in order to maximise the level of glaucoma-induced visual field sensitivity change. ANOVAs were performed for each eye condition (monocular and binocular) and task (pointing and visual localisation) combination.

For completeness, statistical analyses of bias and pointing movement time were performed for both experiments using the same methods as for precision. Negative bias values indicate a bias towards the centre of the screen (inwards), while positive values indicate a bias away from the centre (outwards).

## Results

### Experiment 1

**Figure 3:**
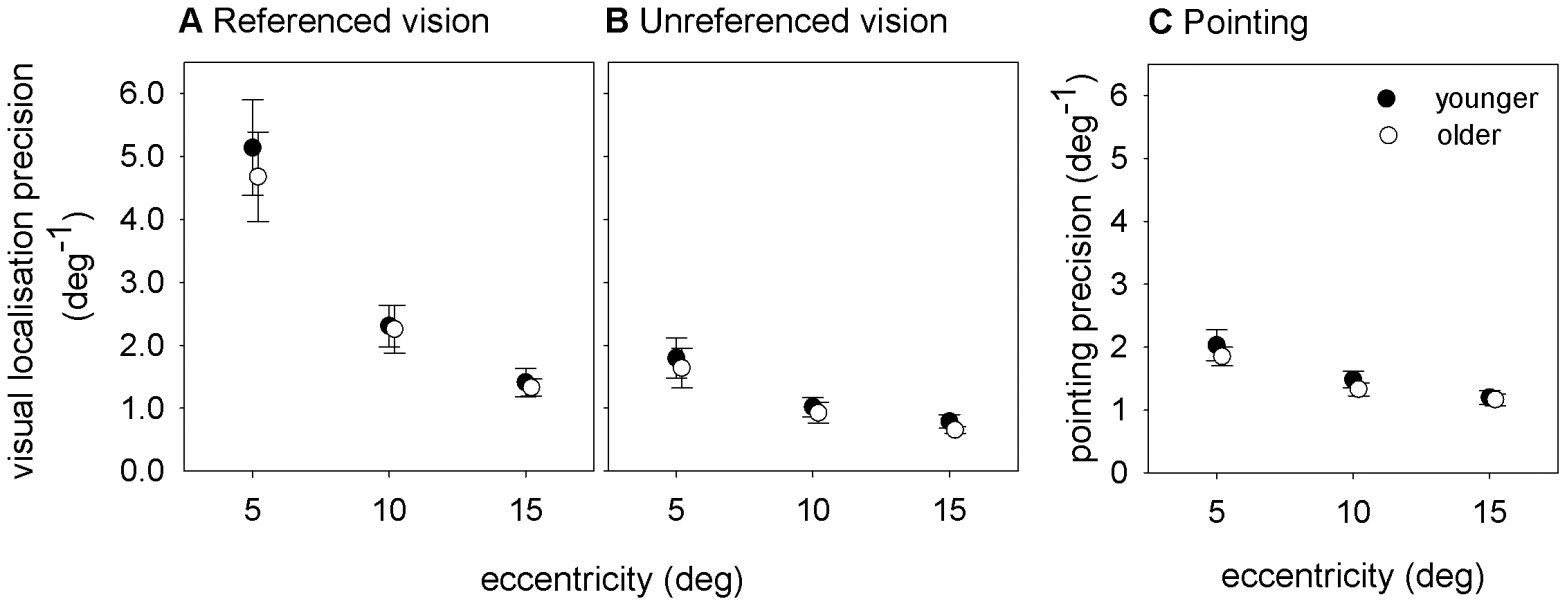
Results of Experiment 1. This plot shows the precision of (A) referenced visual localisation, (B) unreferenced visual localisation and (C) pointing for younger (closed symbols) and older (open symbols) observers at eccentricities of 5°, 10° and 15°. Symbols represent the group mean, with error bars showing 95% confidence intervals for the mean.

Visual localisation precision deteriorated with eccentricity (F(2,94) = 281.7, p<0.001) but was improved by the presence of visual references (F(1,94) = 216.1, p<0.001) (see Figure 3, panels A and B). The older and younger groups had similar levels of task improvement with the presence of visual references (no significant interaction between group and task: F(1,47) = 0.089, p = 0.77) and similar eccentricity performance dependence (no significant interaction between group and eccentricity (F(2,94) = 0.84, p = 0.44)). There was no difference in average group performance between older and younger observers (main effect of group: F(1,47) = 1.01, p = 0.32).

Similar to visual localisation, average pointing precision decreased with eccentricity (F(1,47) = 126.6, p<0.001) and was similar between older and younger observers (no main effect of group: F(1,47) = 2.08, p = 0.16) (see Figure 3C). Movement times were not statistically significantly different for the two age groups when analysed using a repeated measures ANOVA with a within subjects factor of eccentricity (5°, 10° and 15°) and a between subjects factor of group (older and younger) (F(1,48) = 3.77, p = 0.058). Mean movement times for 5°, 10° and 15° were 0.93 s, 0.94 s and 0.99 s, respectively.

Younger and older participants performed with similar degrees of visual localisation bias (F(1,47) = 0.34, p = 0.56), which was on average outwards (mean ±95% confidence interval: 0.24±0.10°). Bias associated with the pointing task was on average inwards, with older adults (mean ±95% confidence interval: −0.79±0.21°) pointing further inwards than younger adults (mean ±95% confidence interval: −0.36±0.21°) (F(1,47) = 8.6, p = 0.005).

**Figure 4:**
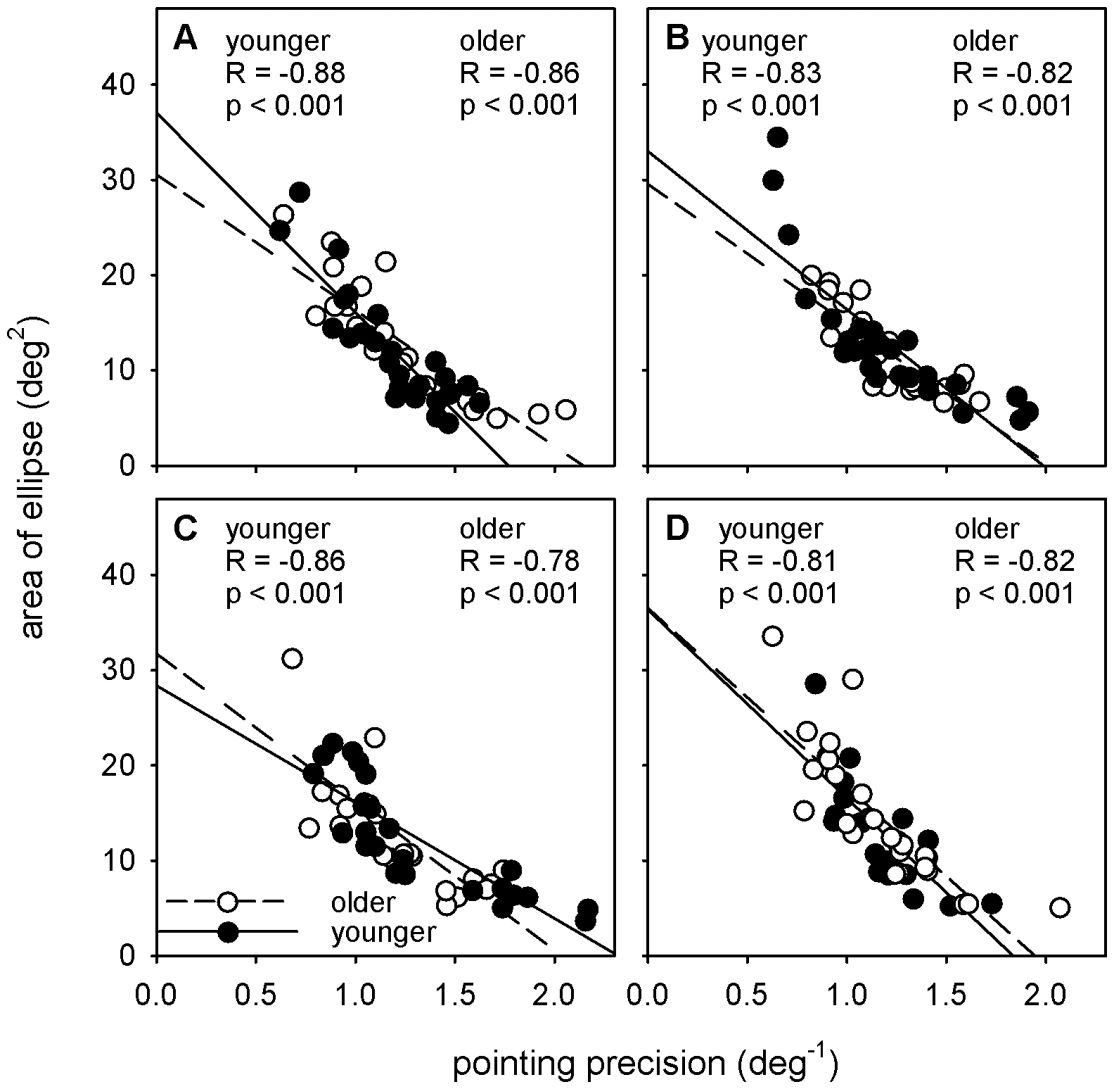
Linear regression of pointing precision against 95% confidence ellipse area for pointing at 15° eccentricity. Data of older participants is represented with open symbols and dashed regression lines, while younger participants are shown with filled symbols and unbroken regression lines. Panels A–D represent the four quadrants that were tested in Experiment 1.

To confirm that radial pointing precision (which relates best to our radial measure of visual localisation) was representative of the overall pointing precision for individuals, we calculated 95% confidence ellipses for the pointing data of each participant. Figure 4 shows a linear regression of ellipse area against radial pointing precision for each of the tested quadrants. Correlations are all strong, with Pearson correlation coefficients in the range of −0.78– −0.88.

Although we expected there to be minimal-to-no learning effect in our data due to observers being familiarised with the task prior to testing, we examined our data from 15° eccentricity to confirm this, being the most difficult task and thus the most likely to show up any difference across trials. For the pointing task we compared the first and the third run. For the visual localisation tasks we compared the results of the first 2 runs to the second 2 runs. Comparisons were done using paired t-tests. There was no statistically significant difference in bias (unreferenced visual localisation: young p = 0.63, old p = 0.52; referenced visual localisation: young p = 0.08, old p = 0.09; pointing: young p = 0.06, old p = 0.24) or precision (unreferenced visual localisation: young p = 0.74, old p = 0.18; referenced visual localisation: young p = 0.56, old p = 0.80; pointing: young p = 0.67, old p = 0.39) for younger and older observers on any of the visual localisation and pointing tasks.

### Experiment 2

The greyscale plots (a greyscale representation of visual field contrast sensitivity in dB relative to age-matched controls, with darker areas indicating poorer sensitivity) of each glaucoma participant, as well as their individual pointing and visual localisation precision, are shown in Figure 2. The right hand panels of Figure 2 suggest that visual field sensitivity minimally affects visual localisation and pointing precision, as most observers have a largely constant level of performance across their range of visual field sensitivities.

**Figure 5:**
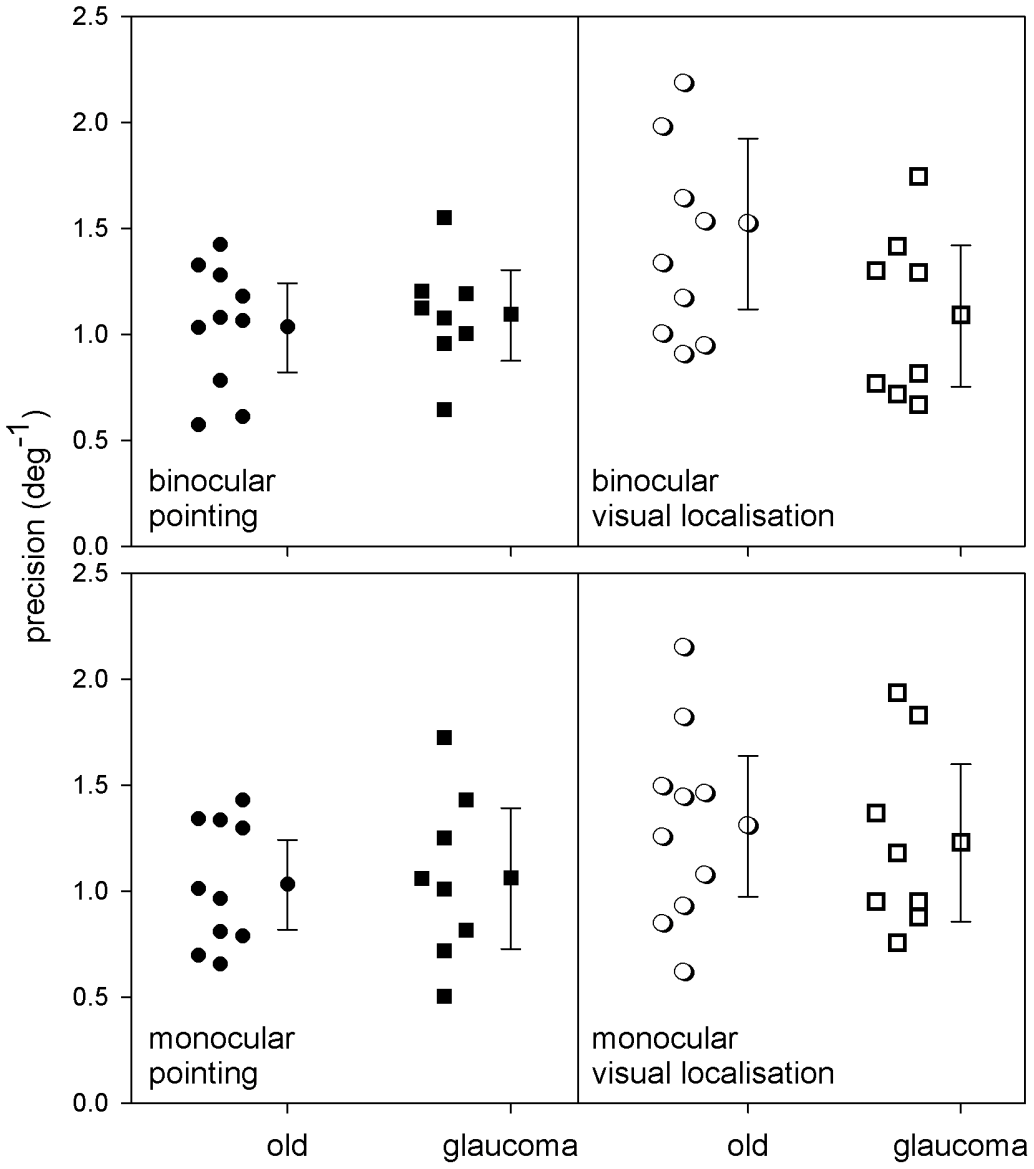
Results of Experiment 2. Pointing and visual localisation precision of glaucoma patients and older controls. Individual data has been plotted next to the mean and 95% confidence interval of the mean for both monocular and binocular conditions.

As predicted from Figure 2 and illustrated in Figure 5, visual localisation and pointing precision did not differ for the older controls and glaucoma patients in either the monocular (visual localisation: *F*(1,16) = 0.14, p = 0.72; pointing: F(1,16) = 0.033, p = 0.86) or binocular (visual localisation: F(1,16) = 3.38, p = 0.085; pointing: F(1,16) = 0.19, p = 0.67) conditions.

There was no difference in bias for the pointing (F(1,16) = 0.17, p = 0.68) or visual localisation (F(1,16) = 0.21, p = 0.66) between the older and glaucoma groups in the binocular condition. However, in the monocular condition, the participants with glaucoma (mean ±95% confidence interval: −0.33±0.44°) visually localised targets closer to fixation than the older controls (mean ±95% confidence interval: 0.31±0.39°) (F(1,16) = 4.81, p = 0.043). It is worth noting that some of our participants had visual field loss closer to fixation and others further from fixation, relative to the position of the presented stimuli, and there was no systematic trend in the bias relative to the location of visual field loss. Interestingly, despite reporting the visual location as closer to fixation, the glaucoma group (mean ± 95% confidence interval: −0.29±0.82°) pointed to locations further from fixation than controls (mean ±95% confidence interval: −2.79±0.75°) on average (F(1,16) = 19.8, p<0.001).

**Figure 6:**
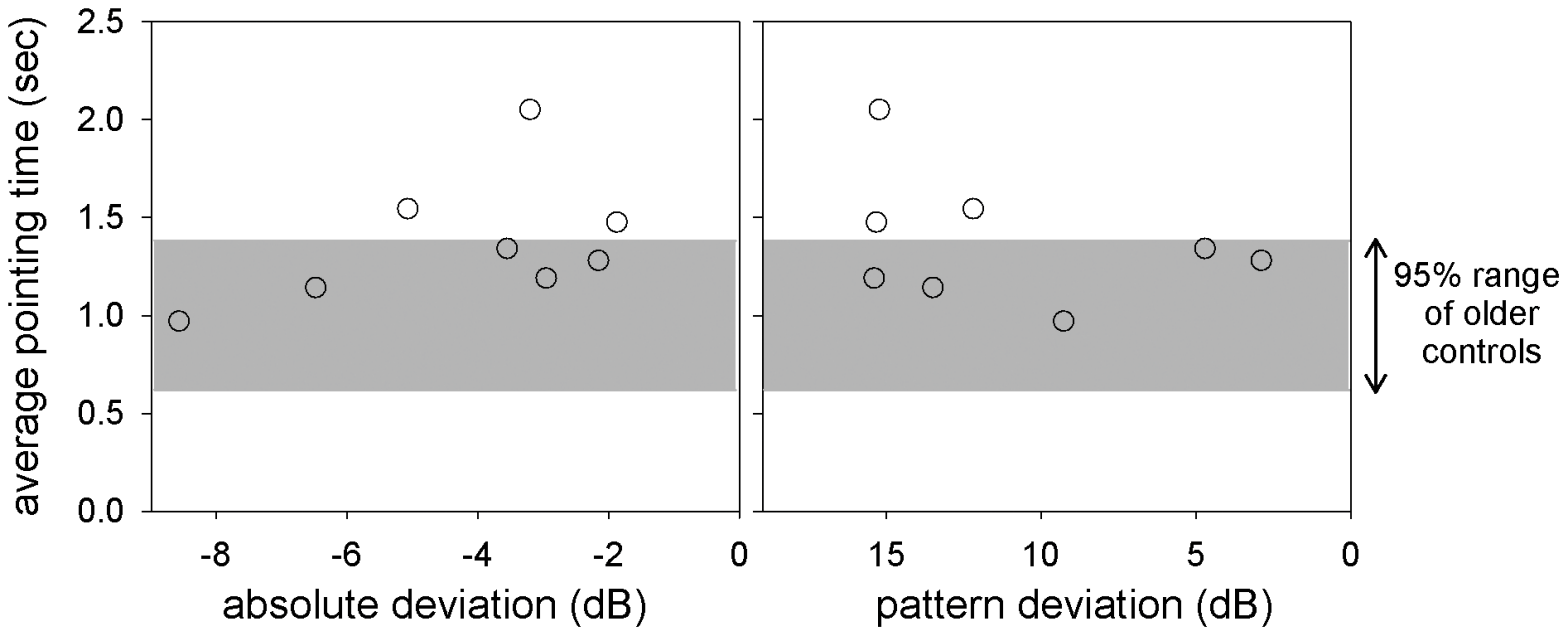
Average pointing time vs. visual field global indices for glaucoma participants. Visual field global indices provide an indication of visual field abnormality relative to an age-matched normative database. Absolute defect status (left panel) is an indication of general visual field loss, with increasingly negative numbers associated with greater levels of visual field loss. Pattern deviation (right panel) describes areas of local visual field loss, with increasingly positive numbers associated with higher levels of localised visual field loss. For both panels data points more to the left indicate greater loss of visual field sensitivity. The grey bands indicate the mean ±2 standard deviations of the older control data.

Participants with glaucoma had statistically significantly longer movement times than older controls when analysed using a repeated measures ANOVA with a within-subject factor of eye condition and a between-subjects factor of group (F(1,16) = 0.293, p = 0.012). Mean movement times for monocular and binocular conditions were: 1.00 s (older) versus 1.37 s (glaucoma) and 1.04 s (older) versus 1.30 s (glaucoma), respectively. Monocular movement times are illustrated in Figure 6.

## Discussion

This study aimed to investigate the relationship between local visual field sensitivity and visuomotor localisation precision in older adults with vision loss. In order to assess the impact of ageing alone, an initial experiment was run on healthy younger and older participants. The results of this initial experiment demonstrated that visual localisation and pointing precision remain largely intact with ageing for highly visible targets presented in the central 15° of the visual field (Figure 3). No significant differences in precision were found when task difficulty was modulated through increasing target eccentricity or removing concurrent visual reference cues. The robustness of visual localisation judgements to ageing is consistent with previous work that shows that positional hyperacuity is minimally altered by ageing both foveally [32], [33], and for peripheral spatial interval discrimination thresholds with ageing [34]. Our findings extend this previous work by showing that non-hyperacuity, non-foveal localisation – arguably a more common daily task – is also unaffected by ageing. Our pointing data is also consistent with a recent report of similar performance of younger and older participants when pointing to a simple target [16], but additionally shows that performance is unimpaired when the location of the visual target is less predictable.

Although auditory feedback was provided for the visual localisation task, no such feedback was given during the pointing task. The rationale for this was that in the visual localisation task there was a clear ‘correct’ and ‘incorrect’ response on which to provide feedback, which was not present in the pointing task. It is possible that providing feedback on the pointing task may have reduced pointing precision. Further study would be required to answer this question.

Our second experiment aimed to investigate the effects of localised visual field loss, assessed with standard automated perimetry, on visual localisation and subsequent pointing precision. We hypothesised that visual localisation precision would deteriorate as visual field sensitivity decreased, an idea that is supported by the impaired planning seen in people with glaucoma during reaching movements [5]. Our results show, however, that there is no difference in visual localisation or pointing precision between glaucoma and age-matched controls under either binocular or monocular viewing conditions for perceived visual stimuli (Figure 5). It must be noted, however, that this result may not be applicable to everyone in the general glaucoma population due to the large variations in visual field defects that characterise this complex disease. Contrary to our expectations, these results suggest that visual localisation and pointing precision to high contrast stimuli in the central 15° of vision remain largely unaffected by reductions in visual field sensitivity, provided that the target is detected. Although the targets were designed to be detected by both groups, in some spatial locations they were closer to threshold due to visual field damage in those with glaucoma. It should also be noted that stimulus durations were shorter than those required to initiate a saccadic eye movement, ensuring that localisation of visual stimuli was performed in the peripheral visual field. It would be interesting to further explore localisation and pointing with lower contrast, natural stimuli, as Wiecek et al [35] found that most participants with glaucomatous visual field loss showed significantly different distribution of eye movement directions during a naturalistic visual search task, providing indirect evidence that peripheral visual field loss may impact visual spatial localisation. Inbuilt redundancies in the visuomotor system may be capable of maintaining performance under suboptimal conditions, either via feedback and feedforward loops, or neural redundancy, allowing the observers with glaucoma to maintain performance. Alternatively, the spatial grain of the visuomotor localisation system may be quite sparse, allowing for significant reductions in spatial content – as might be anticipated from the death of retinal ganglion cells that characterises glaucoma – before visuomotor localisation precision is impacted.

Although the participants with vision loss were able to accurately perform the localisation tasks, the time they required to do so was longer than that of the age matched control group. Our current experiments cannot discern which stage of the visuomotor process requires this additional time; possibly the initial visual spatial localisation coding, the transformation of this visual information into motor plans, or the time taken for the reaching movement itself. It is most likely to be a combination of these, as a prehension study of individuals with glaucoma [5], as well as studies of simulated visual field loss [8], [9], have shown that both the planning and execution of a reach in these subgroups are associated with increased time. The pointing time differences between glaucoma patients and age-matched controls suggest that the visuomotor system may be compensating for the reduced visual field sensitivity and that additional time pressures may result in dysfunction. However, given that movement times were unconstrained, this difference in movement time may also have arisen due to the awareness of glaucoma patients of having reduced vision, or motivational or other psychological factors. Further experiments are needed to explore the performance of the visuomotor system under time stress in older adults with vision loss.

In summary, our results indicate that visual localisation and pointing precision to high contrast stimuli within the central 15° of vision are unaffected by ageing and visual field loss, as occurs in glaucoma. However, these results may not be generalisable to the entire glaucoma population, given the diverse range of visual field deficits that arise from this disease. On average, people with glaucoma did however have a statistically significant increase in their movement time, suggesting that maintaining precision performance in the presence of visual field loss might require this increased duration. Further investigations that increase the demands on the visuomotor system (for example, adding time constraints or reducing the saliency of the stimuli) may help to determine the extent to which the older visuomotor system remains intact in disease and may guide development of technologies, such as tablet computer software, for optimal use by older adults.
